# Systematic Functional Characterization of Human 21st Chromosome Orthologs in *Caenorhabditis elegans*

**DOI:** 10.1534/g3.118.200019

**Published:** 2018-01-24

**Authors:** Sarah K. Nordquist, Sofia R. Smith, Jonathan T. Pierce

**Affiliations:** * Institute for Neuroscience, Department of Neuroscience, The University of Texas at Austin, Texas 78712; † Institute for Cellular and Molecular Biology, Department of Neuroscience, The University of Texas at Austin, Texas 78712; ‡ Center for Learning and Memory, Department of Neuroscience, The University of Texas at Austin, Texas 78712; § Waggoner Center for Alcohol and Addiction Research, Department of Neuroscience, The University of Texas at Austin, Texas 78712

**Keywords:** Down syndrome, neuromuscular, neurological, synaptic

## Abstract

Individuals with Down syndrome have neurological and muscle impairments due to an additional copy of the human 21st chromosome (HSA21). Only a few of ∼200 HSA21 genes encoding proteins have been linked to specific Down syndrome phenotypes, while the remainder are understudied. To identify poorly characterized HSA21 genes required for nervous system function, we studied behavioral phenotypes caused by loss-of-function mutations in conserved HSA21 orthologs in the nematode *Caenorhabditis elegans*. We identified 10 HSA21 orthologs that are required for neuromuscular behaviors: *cle-1* (*COL18A1*), *cysl-2* (*CBS*), *dnsn-1* (*DONSON*), *eva-1* (*EVA1C*), *mtq-2* (*N6ATM1*), *ncam-1* (*NCAM2*), *pad-2* (*POFUT2*), *pdxk-1* (*PDXK*), *rnt-1* (*RUNX1*), and *unc-26* (*SYNJ1*). We also found that three of these genes are required for normal release of the neurotransmitter acetylcholine. This includes a known synaptic gene *unc-26* (*SYNJ1*), as well as uncharacterized genes *pdxk-1* (*PDXK*) and *mtq-2* (*N6ATM1*). As the first systematic functional analysis of HSA21 orthologs, this study may serve as a platform to understand genes that underlie phenotypes associated with Down syndrome.

Down syndrome (DS) is the most common genetic cause of intellectual disability, occurring with an incidence as high as 1 in ∼700 live births in the United States (Canfield *et al.* 2006). Although DS is defined by a variety of symptoms, all individuals with DS display varying degrees of intellectual disability, including learning and memory problems, and most develop Alzheimer-like dementia upon reaching middle age (Coyle *et al.* 1988). In addition, all individuals with DS experience neuromuscular symptoms. For instance, DS is the leading cause of neonatal heart defects, leading to a high infant mortality rate without surgical intervention (Korenberg *et al.* 1994; Vis *et al.* 2009). Another common symptom of DS, hypotonia (muscle weakness), causes deficits in both gross and fine motor skills, causing people with DS to have trouble speaking, writing, and moving efficiently (Pitetti *et al.* 1992).

For over 50 yr, researchers have known that an extra copy of the 21st chromosome (Human somatic autosome 21, HSA21) underlies DS (Jacobs *et al.* 1959). However, the precise mechanisms by which trisomy 21 causes DS-associated phenotypes are largely unknown. An early hypothesis explaining the link between DS and trisomy 21 argued that the burden of the extra genetic material strains cellular processes in the DS patient, resulting in all DS symptoms (Patterson 2009; Shapiro 1975). Yet research on rare individuals with partial trisomy 21 has shown that the amount of extraneous genetic material does not readily account for the collection of symptoms associated with DS nor their degree of manifestation (Korenberg *et al.* 1994; Lyle *et al.* 2009). A second hypothesis posited that a region containing ∼30 protein-coding genes on HSA21, termed the Down Syndrome Critical Region (DSCR), is responsible for all DS-associated phenotypes. This was based on the genomic area of overlap shared between two individuals with partial trisomy 21 who still displayed many DS phenotypes (Rahmani *et al.* 1989). A mouse model (Ts1Rhr) containing trisomy of only DSCR orthologous genes was developed to investigate the role of the DSCR (Olson *et al.* 2004). Many, but not all, of the phenotypes expected for DS were observed in this mouse model, suggesting that the DSCR is important but not solely responsible for the whole set of DS-associated phenotypes (Belichenko *et al.* 2009; Olson *et al.* 2004). Mouse models of DS trisomic for syntenic regions of HSA21 other than the DSCR have also been instrumental in the analysis of the genetic origins of DS-related phenotypes, including craniofacial defects (Richtsmeier *et al.* 2000, 2002), cerebellar cell loss (Baxter *et al.* 2000), as well as synaptic and hippocampal circuit dysfunction (Belichenko *et al.* 2004; Demas *et al.* 1998; Escorihuela *et al.* 1995; Holtzman *et al.* 1996; Reeves *et al.* 1995).

Studies focusing on individual orthologous genes on HSA21 in animal models have also provided insight into the genetic basis of DS phenotypes. Single gene studies are attractive because the underlying genetic contributions of a phenotype are more readily dissected; single genes can also offer clearer insight into pharmacotherapeutic targeting of specific gene products (Dierssen 2012). Determining the biological function of a single gene can be approached with either a loss-of-function or gain-of-function (*e.g.*, overexpression) approach. Both have merit. For genes for which there is no or incomplete redundancy, loss-of-function experiments provide valuable insight into gene function. Several HSA21 orthologous genes were initially characterized in this way in invertebrate models. Early work conducted in *Drosophila melanogaster*, for instance, revealed that the gene *mnb* (*minibrain*) played a critical role in postembryonic neurogenesis (Tejedor *et al.* 1995). Subsequent research linked fly *mnb* to its mammalian ortholog, *DYRK1A*; and, as in fly, loss of *Dyrk1a* in mice was also shown to result in abnormal neurogenesis (Patil *et al.* 1995; Shindoh *et al.* 1996; Song *et al.* 1996, 1997). Intriguingly, overexpression of *Dyrk1a* alone in a mouse transgenic model results in neurodevelopmental delay, motor abnormalities, and spatial memory defects, which suggests a potential role in DS-associated cognitive impairment (Ahn *et al.* 2006; Altafaj *et al.* 2001). Similarly, the neuronal role of the basic helix-loop-helix protein, *SIM2* (single-minded family bHLH transcription factor 2) on HSA21 was also initially identified in fly. Mutations in the fly ortholog, *sim*, impair development of cells in the midline of the central nervous system (Crews *et al.* 1988; Thomas *et al.* 1988). Subsequent experiments identified the mouse homolog and found that a targeted deletion of the gene led to craniofacial malformations (Chen *et al.* 1995; Shamblott *et al.* 2002). Overexpression of *Sim2* in mouse also recapitulated behavioral aspects of established mouse models of DS, including reduced exploratory behaviors and hypersensitivity to pain (Chrast *et al.* 2000). Thus, single gene studies and invertebrate models complement research with traditional DS mouse models that overexpress combinations of many HSA21 genes.

Despite this progress, the *in vivo* function of a majority of genes on HSA21 remains unknown. It is impractical to perform a systematic analysis of gene function through gene knockdown in mouse models. Therefore, we set out to gain knowledge about roles of HSA21 genes by studying the function of HSA21 orthologs in the nematode *Caenorhabditis elegans*.

*C. elegans* is a well-established genetic model and exhibits sequence similarity with at least 42% of genes associated with human disease (Culetto and Sattelle 2000). Additionally, the *C. elegans* nervous system is compact, with only 302 neurons (White *et al.* 1986). Because any one neuron may be connected to any other by only six or fewer synapses, defects in even a single neuron are often detectable with behavioral phenotypes such as locomotion (Chen *et al.* 2006). Proper function of the muscular feeding organ, called a pharynx, also requires precise communication between neurons and muscle. Defects in pharyngeal pumping have helped identify a conserved vesicular glutamate transporter (*e.g.*, *eat-4*) and nicotinic cholinergic receptor subunits (Avery and You 2012). Finally, *C. elegans* synapses are also similar to those of mammals. Genetic screens using the acetylcholinesterase inhibitor aldicarb in worm have identified genes critical for synaptic function in mammals (Richmond 2005).

Because the *in vivo* functions of HSA21 genes have not been systematically studied, we suspected that some HSA21 genes with important neuronal or muscle roles were overlooked. The goal for our study was to determine HSA21 orthologs involved in neuronal and/or muscle function. To this end, we analyzed behavioral phenotypes induced by loss-of-function mutations and found that disruption of several uncharacterized HSA21 orthologs in worm yielded significant neuromuscular phenotypes. To determine if any HSA21 orthologs were involved in neurotransmission, we performed a second screen on loss-of-function mutants measuring their sensitivity to the acetylcholinesterase inhibitor aldicarb. We identified three genes involved in neurotransmitter release: *unc-26* (*SYNJ1*), which has previously been characterized, and *mtq-2* (*N6AMT1*) and *pdxk-1* (*PDXK*), both of which have received minimal attention. The results from our behavioral and pharmacological screens may inform predictions for which genes contribute to the neuronal and muscle phenotypes associated with DS.

## Materials and Methods

### Strains

*C. elegans* were grown on NGM (nematode growth media) agar plates seeded with OP50 bacteria at 20° as described (Brenner 1974). N2 Bristol served as wild type and was also used for outcrossing (Brenner 1974). For a list of outcrossed strains, refer to Supplemental Material, Table S1 (sheet *Worm strains*). The following strains were used is this study: BB3 adr-*2*(*gv42*) III; CB211 *lev-1*(*e211*) IV; CZ18537 *zig-10*(*tm6127*) II; DR97 *unc-26*(*e345*) IV; EK228 *mbk-1*(*pk1389*) X; FX1756 *pad-2*(*tm1756*) III; FX2021 *rcan-1*(*tm2021*) III; FX2626 *Y105E8A.1*(*tm2626*) I; FX2657 *nrd-1*(*tm2657*) I; FX3322 *dnsn-1*(*tm3322*) II; FX3565 *mtq-2*(*tm3565*) II; FX6706 *B0024.15*(*tm6706*) V; FX776 *sod-1*(*tm776*) II; GA503 *sod-5*(*tm1146*) II; MT1072 *egl-4*(*n477*) IV; NS3026 *ikb-1*(*nr2027*) I; PR678 *tax-4*(*p678*) III; PS2627 *dgk-1*(*sy428*) X; RB1489 *D1037.1*(*ok1746*) I; RB2097 *set-29*(*ok2772*) I; RB2436 *cysl-4*(*ok3359*) V; RB2535 *cysl-2*(*ok3516*) II; RB870 *igcm-1*(*ok711*) X; RB899 *cysl-1*(*ok762*) X; RB979 *dip-2*(*ok885*) I; VC200 *rnt-1*(*ok351*) I; VC201 *itsn-1*(*ok268*) IV; VC3037 *mtq-2*(*ok3740*) II; VC40866 *pdxk-1*(*gk855208*) I; VC868 *eva-1*(*ok1133*) I; VC943 *cle-1*(*gk421*) I; VH860 *ncam-1*(*hd49*) X.

### Transgenic strains

We used standard techniques to generate constructs used to make the transgenic strains shown in Table 1.

**Table 1:** Transgenic strains used

JPS610	*vxEx610* [*mtq-2p*::*mtq-2*::*mtq-2 UTR + myo-2p*::*mCherry*::*unc-54 UTR*]; *mtq-2*(*tm3565*)
JPS611	*vxEx611* [*mtq-2p*::*mCherry*::*unc-54 UTR + unc-122p*::*GFP*]
JPS612	*vxEx611* [*mtq-2p*::*mCherry*::*unc-54 UTR + unc-122p*::*GFP*]; *vsIs48* [*unc-17p*::*GFP*]
JPS621	*vxEx621* [*mtq-2p*::*mtq-2*::*mtq-2 UTR + myo-2p*::*mCherry*::*unc-54 UTR*]; *mtq-2*(*tm3565*)
JPS906	*vxEx611* [*mtq-2p:mCherry*::*unc-54 UTR + unc-122p*::*GFP*]; *oxIs12* [*unc-47p*::*GFP + lin-15*(*+*)]
JPS929	*vxEx929* [*pdxk-1p*::*mCherry*::*unc-54 UTR*]
JPS989	*vxEx929* [*pdxk-1p*::*mCherry*::*unc-54 UTR*]; *vsIs48* [*unc-17p*::*GFP*]
JPS990	*vxEx929* [*pdxk-1p*::*mCherry*::*unc-54 UTR*]; *oxIs12* [*unc-47p*::*GFP + lin-15*(*+*)]
JPS1044	*vxEx1044* [*WRM615bF01 + myo-2p*::*mCherry*::*unc-54UTR*]; *B0024.15*(*tm6706*)
JPS1045	*vxEx1045* [*WRM615bF01 + myo-2p*::*mCherry*::*unc-54UTR*]; *B0024.15*(*tm6706*)
JPS1046	*vxEx1046* [*WRM061aD07 + myo-2p*::*mCherry*::*unc-54UTR*]; *rnt-1*(*ok351*)
JPS1047	*vxEx1047* [*WRM061aD07 +myo-2p*::*mCherry*::*unc-54UTR*]; *rnt-1*(*ok351*)
JPS1048	*vxEx1048* [*pdxk-1p*::*pdxk-1*::*pdxk-1UTR*; *myo-2p*::*mCherry*::*unc-54 UTR*]; *pdxk-1*(*gk855208*)
JPS1049	*vxEx1049* [*pdxk-1p*::*pdxk-1*::*pdxk-1UTR*; *myo-2p*::*mCherry*::*unc-54 UTR*]; *pdxk-1*(*gk855208*)
JPS1051	*vxEx1051* [*WRM0611dD03 + myo-2p*::*mCherry*::*unc-54UTR*]; *eva-1*(*ok1133*)
JPS1052	*vxEx1052* [*WRM0611dD03 + myo-2p*::*mCherry*::*unc-54UTR*]; *eva-1*(*ok1133*)
JPS1056	*vxEx1056* [*WRM069bF04 + fat-7p*::*GFP*]; *cle-1*(*gk421*)
JPS1057	*vxEx1057* [*WRM069bF04 + fat-7p*::*GFP*]; *cle-1*(*gk421*)
JPS1058	*vxEx1058* [*WRM0640bC10 + myo-2p*::*mCherry*::*unc-54UTR*]; *pad-2*(*tm1756*)
JPS1059	*vxEx1059* [*WRM0640bC10 + myo-2p*::*mCherry*::*unc-54UTR*]; *pad-2*(*tm1756*)
JPS1060	*vxEx1060* [*WRM0637bA01 + myo-2p*::*mCherry*::*unc-54 UTR*]; *cysl-2*(*ok3516*)
JPS1061	*vxEx1061* [*WRM0637bA01 + myo-2p*::*mCherry*::*unc-54 UTR*]; *cysl-2*(*ok3516*)
JPS1062	*vxEx1062* [*WRM0619dG03 + fat-7p*::*GFP*]; *ncam-1*(*hd49*)
JPS1063	*vxEx1063* [*WRM0619dG03 + fat-7p*::*GFP*]; *ncam-1*(*hd49*)
JPS1064	*vxEx1064* [*cysl-2p*::*mCherry*::*unc-54 UTR*]
JPS1065	*vxEx1065* [*dnsn-1p*::*mCherry*::*unc-54 UTR*]
JPS1066	*vxEx1066* [*WRM0634aG08 + myo-2p*::*mCherry*::*unc-54 UTR*]; *dnsn-1*(*tm3322*)
JPS1067	*vxEx1066* [*WRM0634aG08 + myo-2p*::*mCherry*::*unc-54 UTR*]; *dnsn-1*(*tm3322*)

### RNA interference

We performed RNA interference (RNAi) by feeding as described (Timmons *et al.* 2001). All RNAi clones were obtained from SourceBioScience (Nottingham, UK). First, RNAi-expressing AMP-resistant bacteria were cultured overnight at 37° with shaking in LB broth containing ampicillin (50 mg/ml) to prevent contamination of liquid cultures. The following day, ∼100 μl of bacterial liquid culture was seeded on NGM plates containing 1-mM isopropyl β-D-1-thiogalactopyranoside (IPTG) to induce expression of exogenous RNA by the T7 promoter. Once a bacterial lawn had sufficiently grown, a mixed age population of BZ1272 *nre-1*(*hd20*) *lin-15b*(*hd126*) double-mutant worms was placed in a 2:1 mixture of bleach and 1-M NaOH to kill bacteria and postembryonic worms. This strain was selected due to its heightened sensitivity to RNAi in the nervous system (Schmitz *et al.* 2007). Eggs were allowed to hatch and grow over the next week at 20°, and observed for the following week. This period covers two generations. The first generation of RNAi-treated worms experienced postembryonic effects of the RNAi treatment, while the second generation also experienced maternal and preembryonic effects. We scored viability phenotypes including embryonic lethality, larval lethality, and larval arrest and sterility in either the P0 or F1 generation. Control-treated worms were fed L440 background-strain bacteria that harbored an empty RNAi vector.

### Behavioral assays

All behavioral assays were performed blind to the genotype or RNAi treatment and conducted at room temperature (∼20°).

#### Radial dispersion assay:

To measure radial distance traveled, we placed five to eight d-one adult worms in the center of a 10-cm diameter plate thinly seeded with OP50 bacteria. The distance traveled from the center of the plate was measured at 10 min (Topalidou *et al.* 2017). A minimum of 30 worms were tested per genotype on at least two separate days.

#### Exploration assay:

A single L4-stage worm was placed on a 3.5-cm diameter plate thinly seeded with OP50 bacteria and allowed to crawl freely. After 16 hr, we removed the worm and used the worm track to count how many of 69 squares the explored across. At least 15 worms per genotype were tested on at least two separate days.

#### Aldicarb assay:

Sensitivity to the acetylcholinesterase inhibitor aldicarb was quantified as described (Mahoney *et al.* 2006). At least 25 d-one adult-stage worms were evaluated per trial. For the aldicarb assay using RNAi-treated worms, worms were examined at a single time point (100 min) on 1-mM aldicarb and scored for paralysis. Trials were performed blind to RNAi treatment and in triplicate for two generations (if viable as F1). For the aldicarb assay performed on mutant worms, the number of paralyzed worms on 1-mM aldicarb was noted every half hour for 3 hr. A worm was considered paralyzed when it showed neither spontaneous movement nor movement in response to being prodded three times on the head and tail with a platinum wire. Assays were repeated a minimum of three times.

#### Levamisole assay:

Sensitivity to the acetylcholine receptor agonist levamisole was measured as described (Lewis *et al.* 1980). At least 25 d-one adult-stage worms were placed on plates treated with 800-μM levamisole. We scored worms for paralysis every 10 min for 1 hr. Assays were performed in triplicate.

#### Pharyngeal pumping assay:

We quantified the pumping rate of d-one adult-stage worms by eye for 30 sec under a stereomicroscope at 100× magnification using a handheld counter. A single pump was defined as the backward movement of the grinder (Albertson and Thomson 1976; Raizen *et al.* 1995). At least 30 worms per genotype were analyzed.

### Statistical analysis

For radial dispersion, exploration, and pharyngeal pumping assays, we compared values to the wild-type control with a one-way ANOVA with a Bonferroni correction to evaluate select comparisons or Dunnett’s test when all comparisons were to wild type. Aldicarb and levamisole responses of mutants were compared to wild-type control with a two-way ANOVA and a Bonferroni correction for select comparisons or Dunnett’s test when all comparisons were to wild type. We used GraphPad Prism7 software. To minimize false positives from our screen, we set significance to *P* < 0.001. For all subsequent assays, significance was considered *P* < 0.05.

### Data availability

Strains are available upon request. Table S1, sheet *Worm strains* contains detailed descriptions and sources of all strains used in this study.

## Results

### Estimate of HSA21 protein-coding genes

To determine a conservative number of protein-coding genes on the human 21st chromosome, we queried both Ensembl and Human Gene Nomenclature Committee databases and selected only those proteins reviewed by SwissProt. This public database provides manual curation and review for each protein, which ensures high-quality, nonredundant entries (UniProt Consortium 2014). This strategy yielded a total number of 213 protein-coding genes on the 21st chromosome (see *Orthologs* and *Other Orthologs* sheets in Table S1).

### Determining HSA21 orthologs in C. elegans

**Figure 1:**
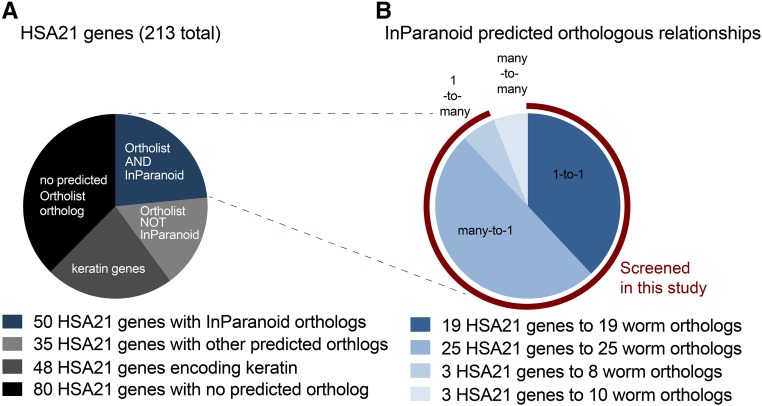
Representation of human 21st chromosome genes in *C. elegans*. (A) HSA21 encodes a conservative estimate of 213 protein-coding genes. Excluding the 48 genes predicted to encode keratin on HSA21 (dark gray), over half of the remaining 165 genes have predicted orthologs in *C. elegans* as identified by OrthoList. All 85 putative orthologs in worm (light gray and blue) with an available RNAi clone were tested in the RNAi viability screen (Table S1). (B) Representation of InParanoid-predicted orthologs with their human to worm relationship (shades of blue). We tested all predicted InParanoid orthologs except those within a many-to-many relationship with RNAi for viability and mutants for behavioral and aldicarb assays (red).

To identify putative worm orthologs of protein-coding genes on the human 21st chromosome (HSA21), we relied on OrthoList, a publicly available database. OrthoList compiles results from a meta-analysis of four orthology prediction programs—Ensembl Compara, OrthoMCL, InParanoid, and Homologene (Shaye and Greenwald 2011). OrthoList predicts at least one worm ortholog for 85 of the 213 HSA21 protein-coding genes (blue and light-gray wedges in Figure 1A). HSA21 encodes 48 predicted keratin proteins, which have no orthologs in worm (Shaye and Greenwald 2011). Excluding these keratin-encoding genes, *C. elegans* has at least one predicted orthologous gene to 52% of the remaining HSA21 genes (Figure 1A). To focus our screen on genes that most likely share conserved functions, we chose to study orthologous genes existing in any relationship except many-to-many as defined by the InParanoid algorithm (Sonnhammer and Östlund 2015). This included one-to-one, one-to-many, and many-to-one relationships. By this measure, InParanoid identifies 47 unique HSA21 genes with predicted orthologs in worm (three wedges highlighted by red arc in Figure 1B). These 47 HSA21 human genes are represented by 51 genes in worm.

The imperfect evolutionary matchup of HSA21 human genes with *C. elegans* genes results in many human genes being orthologous to multiple worm genes (Figure 1B and see *Orthologs* sheet in Table S1). For instance, the human gene *CBS* is predicted to be orthologous to four paralogs: *cysl-1*, *cysl-2*, *cysl-3*, and *cysl-4*. There was only one case of paralogous human genes in our analysis: both *RRP1* and *RRP1B* human genes are predicted to be orthologous to the single worm gene *C47E12.7*. Of the 47 HSA21 human genes, 19 are orthologous one-to-one with worm genes, 25 are orthologous many-to-one with 25 worm genes, and three are orthologous one-to-many with eight worm genes.

An additional 35 HSA21 human genes had one or more orthologs predicted by the OrthoList algorithm in *C. elegans* (depicted by light-gray wedge in Figure 1A and listed in *Other Orthologs* sheet in Table S1). Because most of these HSA21 human genes are predicted to be orthologous to multiple worm genes, the orthology of the 35 human genes expands to represent 111 worm genes in total. For example, the human gene *ABCG1* is predicted to be orthologous to four paralogs: *wht-2*, *wht-3*, *wht-6*, and *wht-8*. In four cases, distinct but paralogous human genes are orthologous to the same worm gene. For instance, both *OLIG1* and *OLIG2* human genes are both predicted to be orthologous to *hlh-16* in worm. Due to the divergent nature of these orthologies, these 111 orthologs in worm are less likely to represent functional orthologs of HSA21 human genes. Therefore, we did not pursue them in this study.

Lastly, we found that an additional 128 protein-coding genes on HSA21 have no predicted OrthoList ortholog in *C. elegans* (depicted by black wedge in Figure 1A and listed in *No Orthologs* sheet in Table S1).

Taken together, our analysis above shows that discounting the keratin genes, about half of the 213 HSA21 human genes are predicted to be orthologous to genes in *C. elegans*. We focused on the set of 47 HSA21 human genes represented by 51 InParanoid orthologs in worm in this study.

### HSA21 orthologs required for viability in C. elegans

Before embarking on our behavioral screen using mutants, we first sought to determine which of the 51 HSA21 predicted orthologs are required for viability using RNAi (see *Orthologs* sheet in Table S1). Previous large-scale and small-scale RNAi screens studied 38 of the 51 genes (Ceron *et al.* 2007; Cui *et al.* 2008; Fraser *et al.* 2000; Gottschalk *et al.* 2005; Kamath *et al.* 2003; Maeda *et al.* 2001; Simmer *et al.* 2003; Sönnichsen *et al.* 2005). These studies reported that RNAi treatments caused viability phenotypes for 15 of these orthologs that severely effect development, growth, and/or reproduction. These include embryonic and larval lethal, larval arrest, severe growth defects, and sterility phenotypes. For an additional 24 orthologs, these studies reported that RNAi treatment caused an apparent wild-type phenotype. Out of the 51 HSA21 worm orthologs, 14 were not studied in previous RNAi screens, in part, because no RNAi reagent was available at the time.

To confirm and extend these results we performed RNAi on all 51 HSA21 orthologs for which an RNAi clone was currently available using 45 unique RNAi treatments (see *Orthologs* sheet in Table S1). We found concordance for 82% or 37 out of the 45 genes tested. Only 4% or two of our RNAi treatments produced results discrepant from previous studies. RNAi treatment of *irk-1* (*KCNJ6*) or *chaf-2* (*CHAF1B*) were previously reported to cause severe viability phenotypes whereas we found no effect for both treatments (Gottschalk *et al.* 2005; Kamath *et al.* 2003; Nakano *et al.* 2011; Sönnichsen *et al.* 2005). We found that 14 out of 16 of the genes that caused inviability in our study or in previous studies when knocked down, including *irk-1* and *chaf-2*, lacked a publicly available viable loss-of-function mutant. This finding is consistent with the idea that these genes are probably essential. Two additional genes, *dip-2* and *dnsn-1*, had corresponding viable predicted null mutants, but we and others found that they caused inviability when knocked down by RNAi (Kamath *et al.* 2003; Simmer *et al.* 2003); perhaps this reflects a viability phenotype with low penetrance. We acknowledge that additional genes may later prove to represent essential genes too, especially if the gene lacks a corresponding null mutant, *e.g.*, *pdxk-1*. Thus, overall our RNAi screen confirms most viability phenotype results from previous studies and suggests that at least 33% or 15 out of 45 of tested HSA21 predicted orthologs are inviable when reduced in function in *C. elegans*.

### HSA21 orthologs required for neuromuscular behaviors

Having identified which HSA21 orthologs are probably essential, we turned next to investigate the *in vivo* function of nonessential HSA21 orthologs. We tested every publicly available, viable mutant, which represented 27 genes in total (see *Orthologs* sheet in Table S1). We performed a phenotypic screen, selecting a battery of three behavioral assays to probe neuromuscular function. Overall, we identified 10 mutants that were significantly defective in performance in these assays. To better link genotype to phenotype, we generated transgenic rescue lines for any mutant deficient in any of the three behaviors and/or tested additional mutant alleles if available.

**Figure 2:**
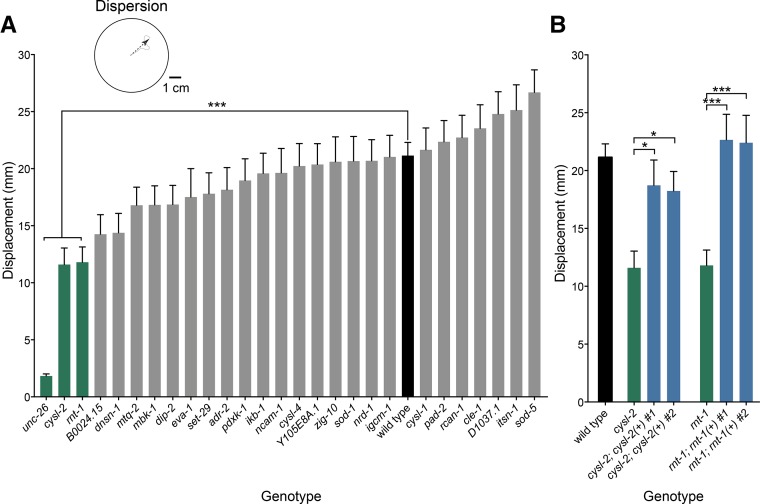
Screen for radial dispersion defects. Histograms show the mean displacement ± SEM from start over 10 min for each worm during a radial dispersion assay. (A) Three mutants (green) showed substantial reductions in dispersal relative to wild type (black). *** *P* < 0.001, *n* > 30. (B) Reductions in dispersal were rescued for the *cysl-2* and *mt-1* mutants (green) by extrachromosomal expression of the wild-type genes (blue). Wild-type performance shown for comparison (black). * *P* < 0.05, ** *P* < 0.01, *** *P* < 0.001, *n* > 30.

We first examined short-term locomotion with a radial dispersion assay, which provides a general metric of locomotor function over 10 min. In this assay, the distance displaced over time reflects a combination of locomotor traits including frequency of body bends, reversal rate, and efficient coordination (Topalidou *et al.* 2017). Aside from the well-characterized uncoordinated mutant *unc-26*, an ortholog of synaptojanin (Harris *et al.* 2000), we also identified two other mutants with rescuable deficits in radial dispersion: *cysl-2* (*CBS*) and *rnt-1* (*RUNX1*) (Figure 2).

**Figure 3:**
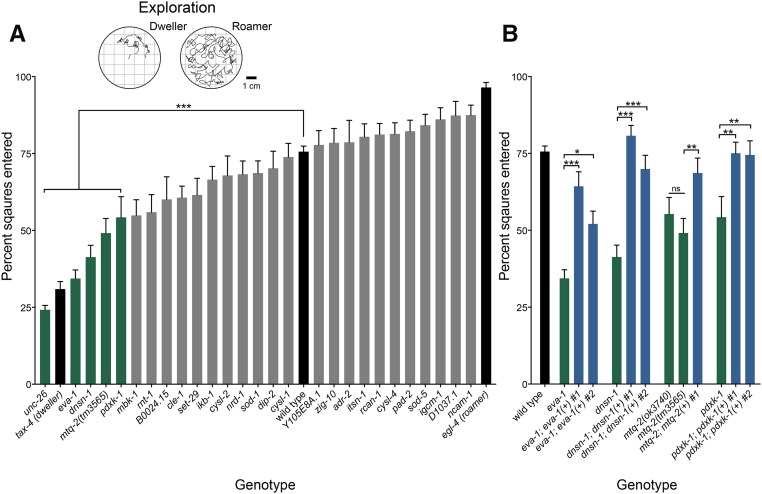
Screen for exploration defects. Histograms show the mean percentage of the assay plate traversed (percent of squares entered ± SEM) by individual worms over a 16-hr exploration assay. (A) Five mutants (green) covered significantly less area than wild type (black). *** *P* < 0.001, *n* > 15. (B) Reductions in exploration were rescued in the *eva-1*, *C24H12.5*, *mtq-2*(*tm3565*), and *pdxk-1* mutants (green) by extrachromosomal expression of each of the wild-type genes (blue). Wild-type performance shown for comparison (black). * *P* < 0.05, ** *P* < 0.01, *** *P* < 0.001, *n* >15.

Second, we examined the longer-term behavior of mutants with an exploration assay. This assay measures not only the general locomotor ability of worms, but also their tendency to explore a cultivation plate seeded with bacterial food over the course of 16 hr. While foraging, worms alternate between two states: roaming, an active state characterized by a burst of locomotion associated with a search for food; and dwelling, a more passive state associated with feeding or resting after satiation. Time spent in roaming and dwelling states depends on integrating internal neuromodulatory cues with external sensory cues. The absence of such sensory transduction leads to extended dwelling as observed in the *tax-4* mutant, which encodes a cyclic nucleotide-gated channel subunit (Fujiwara *et al.* 2002; Greene *et al.* 2016). In contrast, mutants with constitutive sensory input, such as the *egl-4* mutant, show extended roaming (Fujiwara *et al.* 2002). Extended dwelling or roaming compared to wild-type behavior can be quantified by counting the number of squares that a worm traverses over 16 hr (Figure 3).

As expected, we found that the uncoordinated mutant *unc-26* (*SYNJ*) moved poorly in the exploration assay. We also identified the following four mutants with rescuable defects in extended dwelling: *eva-1* (*EVA1C*), *dnsn-1* (*DONSON*), *mtq-2* (*N6AMT1*), and *pdxk-1* (*PDXK1*) (Figure 3A). These mutants appeared to move superficially similar to wild type, suggesting sensory-motor integration deficits. We rescued the exploration behavior of each mutant by transformation with wild-type copies of corresponding transgenes (Figure 3B).

**Figure 4:**
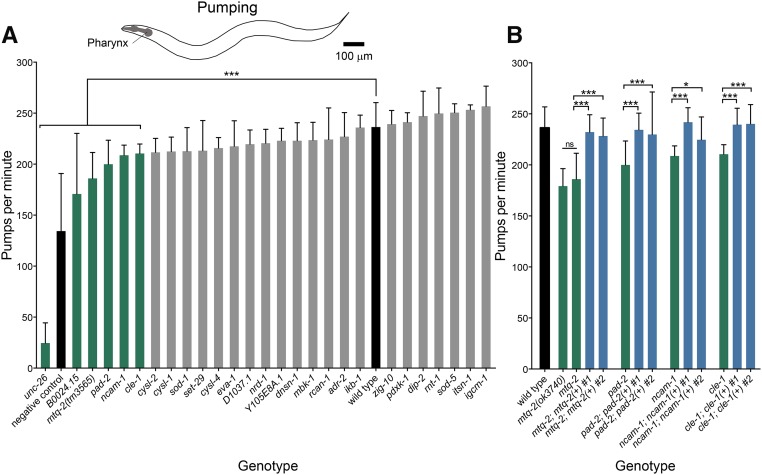
Screen for pumping defects. Histograms showing mean pharyngeal pumps per minute (±SD) in the presence of standard bacterial food. (A) Seven mutants (green) showed significant defects in pharyngeal pumping relative to wild type (black). *** *P* < 0.001, *n* > 30. (B) Mutants with pharyngeal pumping defects *mtq-2*(*tm3565*), *pad-2*, *ncam-1*, and *cle-1* (green) were rescued by extrachromosomal expression of the wild-type genes (blue). We did not observe rescue of *B0024.15*. * *P* < 0.05, *** *P* < 0.001, *n* > 30.

To obtain a measure of neuromuscular activity that was distinct from locomotion, we performed a feeding assay. *C. elegans* pumps bacterial food into its gut through a feeding organ called a pharynx that uses muscles and neurons distinct from those needed for locomotion. The rhythmic contractions and relaxations of the pharynx require precise coordination between pharyngeal neurons and muscle. We identified the following four genes required for normal pharyngeal pumping: *cle-1* (*COL18A1*), *mtq-2* (*N6AMT1*), *ncam-1* (*NCAM2*), and *pad-2* (*POFUT2*) (Figure 4A). Independent null alleles of *mtq-2* displayed similar deficits in pharyngeal pumping (Figure 4B). Transformation with wild-type copies of each gene rescued the slow pumping phenotype of each mutant (Figure 4B).

In summary, by screening three behaviors, we identified 10 genes total with rescuable defects in one or more behaviors designed to probe neuromuscular function. Several among these genes—*dnsn-1* (*DONSON*), *pdxk-1* (*PDXK*), and *mtq-2* (*N6AMT1*)—are poorly characterized, yet may mediate nervous system function, development, or both.

### HSA21 orthologs required for proper synaptic function

To determine the potential role of HSA21 predicted orthologs in neurotransmission, we tested whether the same set of loss-of-function mutants displayed altered sensitivity to paralysis by the acetylcholinesterase inhibitor aldicarb. Many genes critical for key steps in synaptic function have been identified by testing mutants for altered sensitivity to aldicarb (Richmond 2005). Aldicarb causes paralysis of worms by chronically activating body-wall muscle after prolonged presence of acetylcholine at the neuromuscular junction. Mutants defective in any aspect of synaptic transmission show altered sensitivity to paralysis by aldicarb. For instance, mutants defective in acetylcholine release show resistance to paralysis by aldicarb due to decreased levels of acetylcholine in the cleft (Mahoney *et al.* 2006). Conversely, mutations that increase acetylcholine release lead to increased sensitivity to paralysis (Gracheva *et al.* 2006; McEwen *et al.* 2006).

**Figure 5:**
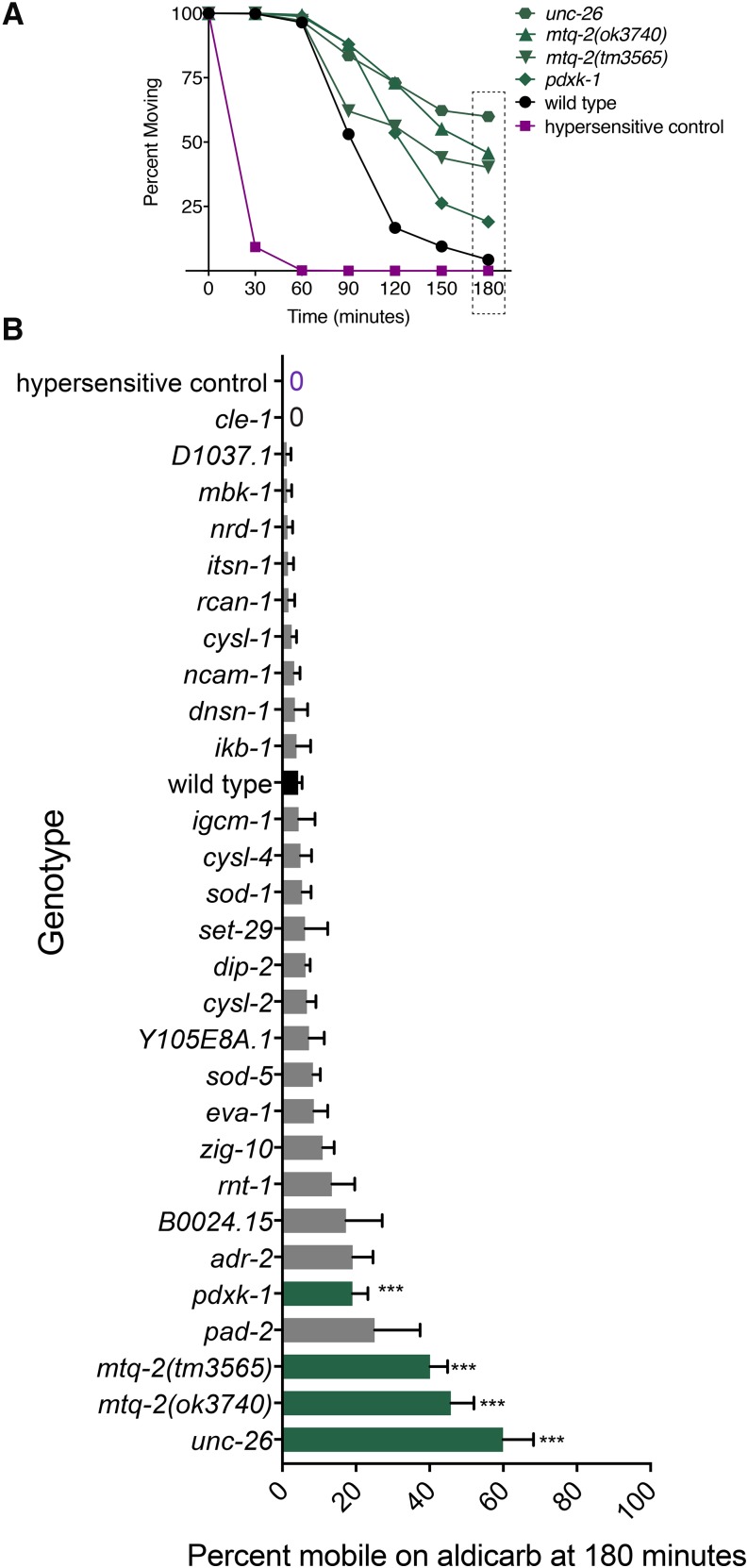
Screen for aldicarb resistance. We measured the full 180-min time course to paralysis for 28 strains including wild type (WT) and hypersensitive control (*dgk-1*) in 1-mM aldicarb. Significant difference in paralysis rate was determined from WT with a two-way ANOVA using Dunnett’s correction for multiple comparisons. No mutants showed significant hypersensitivity. (A) Mean percent (±SEM) of animals moving on aldicarb over minutes for WT, positive control, and mutants that were significantly different from WT: *pdxk-1*(*gk855208*), *mtq-2*(*tm3565*), *mtq-2*(*ok3740*), and *unc-26*(*e345*). *n* > 3. (B) Mean percent of (±SEM) of animals moving on aldicarb at the 180 min time point for all mutants tested including those (green) that were significantly different from WT (black). Zero indicates that no animals were moving. *** *P* < 0.001.

**Figure 6:**
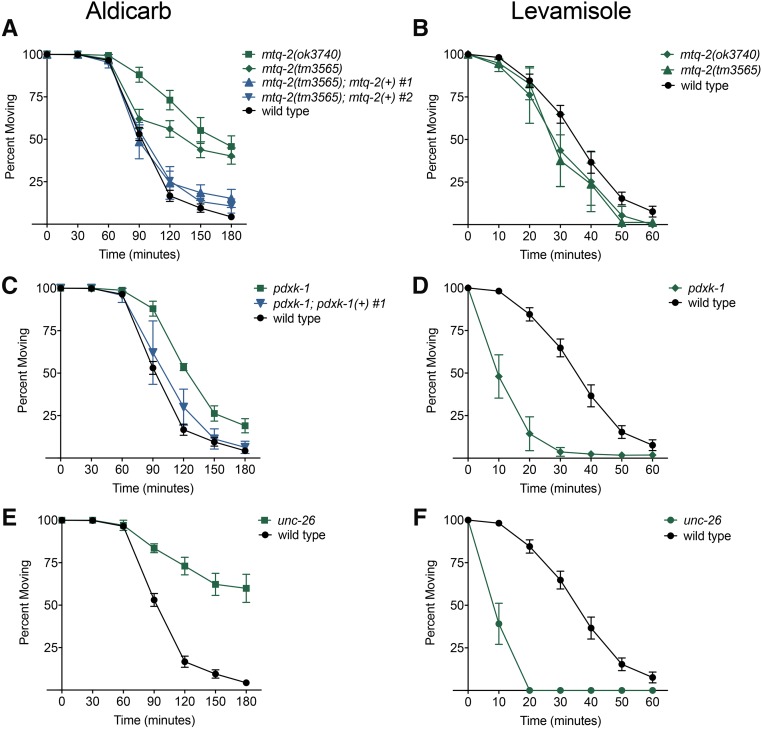
Response of deletion and putative loss-of-function mutants to aldicarb and levamisole. Time course to paralysis on 1 mM aldicarb or 800 μM levamisole for aldicarb-resistant mutants identified in screen (*n* > 3 trials, ∼25 worms per trial). Wild-type controls were assayed in parallel. (A) Two independent alleles of *mtq-2* (green) displayed significant resistance to aldicarb, *P* < 0.001. Two of two rescue lines of *mtq-2*(*tm3565*) (blue) displayed significant sensitivity from the mutant background, *P* < 0.05. (B) Two independent alleles of *mtq-2* displayed sensitivity to levamisole. (C). *pdxk-1*(*gk855208*) displayed significant resistance to aldicarb, *P* < 0.001. One of two rescue lines of *pdxk-1*(*gk855208*) (blue) was significantly improved from the mutant background, *P* < 0.01. (D) *pdxk-1*(*gk855208*) displayed hypersensitivity to levamisole relative to wild type, *P* < 0.001. (E) *unc-26*(*e345*) displayed significant resistance to aldicarb, *P* < 0.001 and (F) hypersensitivity to levamisole, *P* < 0.001.

We identified three mutants that displayed significant resistance to paralysis by aldicarb: *unc-26* (*SYNJ1*), *mtq-2* (*N6AMT1*), and *pdxk-1* (*PDXK*). Compared to wild type, these three mutant strains took longer to paralyze when treated with aldicarb (Figure 5A and Figure 6). By contrast, 24 other mutants became paralyzed at a similar rate as wild-type worms. Although we measured the 180-min time course of paralysis for all 27 mutants, we plot only the percent moving on aldicarb at the 180-min time point for ease of comparison (Figure 5B). Transformation of *mtq-2* and *pdxk-1* mutants with wild-type copies of these genes rescued aldicarb sensitivity for each mutant (Figure 6). As a complementary strategy to the mutant screen, we also tested aldicarb sensitivity for HSA21 predicted orthologs using RNAi. With this independent approach, we confirmed the aldicarb resistance of *pdxk-1* (*PDXK-1*) and observed suggestive results for *mtq-2* (*MTQ-2*) (Figure S1).

We found that RNAi treatment targeting *ncam-1* provided resistance to aldicarb; however, we also found that a null allele of *ncam-1* failed to show any resistance to paralysis by aldicarb (Figure 4B and Figure 5), suggesting that our RNAi result for *ncam-1* was a false positive. This result is consistent with a previous RNAi study that found negative results for *ncam-1* on aldicarb (Sieburth *et al.* 2005).

Several decades of studies have concluded that resistance to aldicarb is primarily caused by presynaptic deficiencies in acetylcholine release or postsynaptic deficiencies in receptor response (Mahoney *et al.* 2006). To distinguish between these possibilities, we measured the sensitivity of mutants to paralysis by levamisole, a potent agonist for the nicotinic cholinergic receptor (Lewis *et al.* 1980). Levamisole causes paralysis of wild-type worms by chronically activating body-wall muscle irrespective of synaptic release defects. Mutants resistant to paralysis by levamisole are defective in cholinergic receptor signaling or muscle function.

We found that *mtq-2*, *unc-26*, and *pdxk-1* mutants all displayed wild-type-like or hypersensitive responses to levamisole (Figure 6). These results suggest that resistance to aldicarb among these three mutants is due to defective presynaptic release of neurotransmitter.

### Expression pattern of HSA21 orthologs

**Figure 7:**
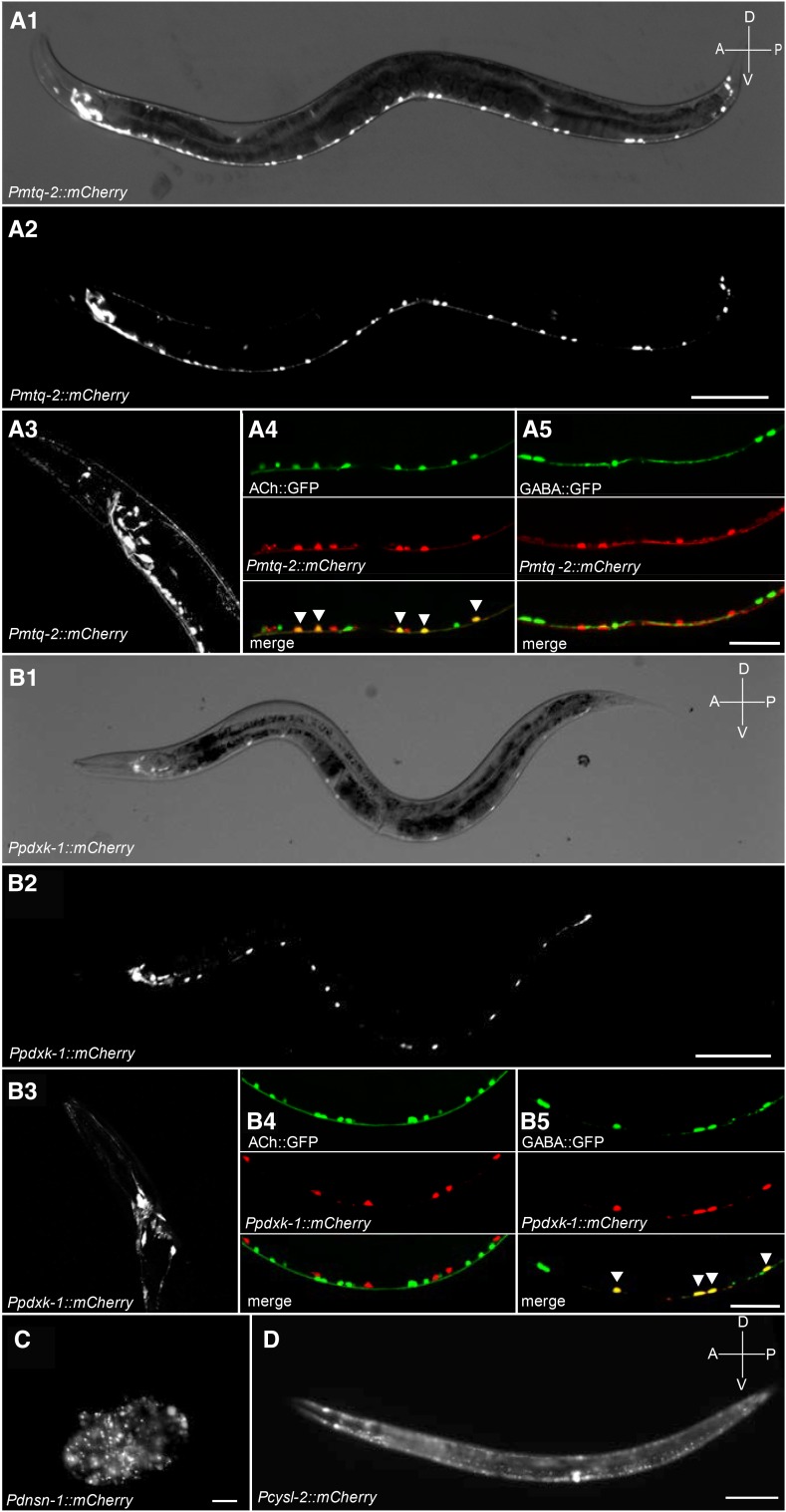
Expression pattern of select genes. Fluorescent micrographs of all HSA21 ortholog hits for which expression patterns were unknown. (A1–A3) A transcriptional reporter for *mtq-2* expressed mCherry throughout the adult nervous system including in head (A3). Bright field image shown in (A1) for reference. Scale bar, 100 μm. Dual color images show coexpression of *mtq-2* reporter in cholinergic (arrowheads, A4) but not in GABAergic neurons (A5) in the ventral nerve cord. Scale bar, 50 μm. (B1–B3) A transcriptional reporter for *pdxk-1* expressed mCherry throughout the adult nervous system including in head (B3). Bright field image shown in B1 for reference. Scale bar, 100 μm. Dual color images show coexpression of *pdxk-1* reporter in GABAergic neurons (B5) but not cholinergic neurons (B4) in the ventral nerve cord. Scale bar, 50 μm. (C) A transcriptional reporter for *dnsn-1* expressed mCherry widely in developing embryo. Scale bar, 50 μm. (D) A transcriptional reporter for *cysl-2* expressed mCherry throughout body-wall and vulval muscle. Scale bar, 100 μm.

We identified nine genes linked to rescuable neuromuscular defects in our study (Table 2). Five genes were previously shown to be expressed in the nervous system: *cle-1* (*COL18A1*), *eva-1* (*EVA1C*), *ncam-1* (*NCAM2*), *pad-2* (*POFUT2*), *rnt-1* (RUNX1), and *unc-26* (*SYNJ*) (Ackley *et al.* 2001; Fujisawa *et al.* 2007; Hunt-Newbury *et al.* 2007; McKay *et al.* 2003; Menzel *et al.* 2004; Schwarz *et al.* 2009). An additional four genes had no reported expression patterns: *cysl-2* (*CBS*), *dnsn-1* (*DONSON*), *mtq-2* (*N6AMT1*), and *pdxk-1* (*PDXK*). To identify the tissues in which these genes may function, we generated mCherry transcriptional reporter strains. We found that *mtq-2* expressed exclusively throughout the nervous system. Expression overlapped with a subset of cholinergic neurons in the head and throughout the ventral nerve cord as defined by the *Punc-17*::*GFP* reporter (Figure 7A4). Expression of *mtq-2* mCherry reporter was not observed in GABAergic neurons defined by the *Punc-47:GFP* reporter (Figure 7A5). Interestingly, our mCherry reporter for *pdxk-1* expressed in GABAergic, but not cholinergic neurons (Figure 7B). Our transcriptional reporter for *dnsn-1* expressed robustly within the developing embryo, but not within the adult nervous system (Figure 7C). This suggests a potential developmental role for this gene. Lastly, we found that our mCherry *cysl-2* reporter expressed broadly throughout head and body-wall muscle (Figure 7D).

**Table 2:** Summary of HSA21 gene orthologs required for normal behaviors in *C. elegans*

Gene	Description	Behaviors				Expression			HSA21 Gene*^a^*	Mouse Gene*^a^*	Mouse Viability*^b^*
		Dispersion	Exploration	Pumping	Aldicarb	Neuron	Muscle	Other			
*cle-1*	Collagen			↓		✓			*COL18A1*	*Col18a1*	Viable
*cysl-2*	Cysteine synthase	**↓**					✓	✓	*CBS*	*Cbs*	Postnatal lethal*^c^*
*dnsn-1*	Novel		↓					✓	*DONSON*	*Donson*	Viable
*eva-1*	Slit receptor		↓			✓			*EVA1C*	*Eva1c*	Viable
*mtq-2*	Glutamine methyltransferase		↓	↓	RIC	✓			*N6AMT1*	*N6amt1*	Embryonic lethal*^d^*
*ncam-1*	Neural cell adhesion molecule			↓		✓			*NCAM2*	*Ncam2*	Viable
*pad-2*	O-fucosyltransferase			↓		✓	✓		*POFUT2*	*Pofut2*	Preweaning lethal*^e^*,*^f^*
*pdxk-1*	Pyridoxal kinase		↓		RIC	✓			*PDXK*	*Pdxk*	Preweaning lethal
*rnt-1*	Runx transcription factor	↓				✓	✓	✓	*RUNX1*	*Runx1*	Embryonic lethal*^e^*
*unc-26*	Synaptojanin	↓	↓	↓	RIC	✓			*SYNJ1*	*Synj1*	Perinatal lethal*^f^*

↓ indicates the direction of changes that significantly differ from wild type (*P* < 0.001, one-way ANOVA). RIC (Resistant to Inhibitors of Cholinesterase) indicates mutants with significant resistance to aldicarb *vs.* wild type (*P* < 0.001, two-way ANOVA). ✓ indicates tissue(s) in which the gene is expressed. Data were derived from WormBase.*^g^* Underlining signifies expression results found in this study.

a HSA21 gene and mouse gene orthologs derived from InParanoid.*^h^*

b Mouse viability data derived from the Mouse Genome Informatics database.*^i^* Specific citations are indicated with a footnote.

c Watanabe *et al.*, 1995.

d Liu *et al.*, 2010.

e Okuda *et al.*, 1996.

f Cremona *et al.*, 1999.

g Lee *et al.*, 2017.

h Sonnhammer and Östlund, 2015.

i Blake *et al.*, 2017.

## Discussion

Our research predicts new functional annotation of genes on the human 21st chromosome by characterizing *in vivo* roles of putative orthologs in *C. elegans*. Within the field of DS, research has primarily focused on the functions of contiguous groups of genes using mice, such as those included in the so-called DSCR (Jiang *et al.* 2015). Meanwhile, the vast majority of HSA21 genes remain functionally uncharacterized. One proven approach to reveal the function of large numbers of genes, even at the level of the whole genome, is to study their roles in more tractable invertebrate models such as *C. elegans*. Although several large-scale reverse genetics screens in *C. elegans* have begun to uncover novel *in vivo* roles for thousands of genes, they have focused less explicitly on neuronal phenotypes and excluded more genes than may be generally acknowledged (Fraser *et al.* 2000; Kamath *et al.* 2003; Sieburth *et al.* 2005; Vashlishan *et al.* 2008). For instance, two seminal whole-genome RNAi screens tested only 33 of the 51 HSA21 orthologs focused on in this study (Fraser *et al.* 2000; Kamath *et al.* 2003). These studies primarily focused on viability phenotypes such as gross development and fertility. We expanded this analysis to test an additional eight orthologs not tested in these seminal or subsequent RNAi studies (Ceron *et al.* 2007; Gottschalk *et al.* 2005; Nakano *et al.* 2011; Pujol *et al.* 2001; Rual *et al.* 2004; Sönnichsen *et al.* 2005). With the addition of our current study, out of the 51 HSA21 orthologs, only five genes remain unstudied using RNAi or mutant in any study due to lack of reagents: *D1086.9* (*CLIC6*), *H39E23.3* (*CLIC6*), *Y54E10A.11* (*LTN1*), *Y74C10AL.2* (*TMEM50B*), and *ubc-14* (*UBE2G2*). Overall, however, our results were consistent with earlier studies. We found only one discrepancy, where a previous study found that RNAi targeting *irk-2* caused sterility while we found it had no effect (Kamath *et al.* 2003).

Other seminal RNAi screens in *C. elegans* searched for genes critical for synaptic transmission. Even though these screens were prodigious, testing 2068 genes in total, they only tested five of the HSA21 orthologs: *mbk-1*, *itsn-1*, *ncam-1*, *K02C4.3*, and *wdr-4* (Sieburth *et al.* 2005; Vashlishan *et al.* 2008). These studies reported negative results for all five genes in agreement with our RNAi and mutant findings here. Notably, the synaptic RNAi screens did not test the three genes that we found to be critical for normal synaptic transmission: *mtq-2*, *pdxk-1*, and *unc-26*. Thus, despite the whole genome and large-scale nature of previous screens, many genes with important *in vivo* functions likely remain to be discovered using *C. elegans*. Because our study largely replicated results from previous studies, however, we are optimistic that a systematic approach with *C. elegans*, as used here, may reveal the function of important sets of genes involved in human polygenic or chromosomal disorders.

We also performed several behavioral screens to identify HSA21 predicted orthologs that function in nervous system and muscle in ways that previous reverse-genetic screens may have missed. This included a radial dispersion assay that reflects short-term locomotor ability, an exploration assay that reflects longer-term sensory and motor integration, and a feeding assay that probes an independent neuromuscular circuit. We identified 10 mutants with deficiencies in these behaviors that we were able to rescue and/or confirm with independent alleles (Table 2): *cle-1* (*COL18A1*), *cysl-2* (*CBS*), *dnsn-1* (*DONSON*), *eva-1* (*EVA1C*), *mtq-2* (*N6AMT1*), *ncam-1* (*NCAM2*), *pad-2* (*POFUT2*), *pdxk-1* (*PDXK*), *rnt-1* (*RUNX1*), and *unc-26* (*SYNJ*). These 10 orthologs represent alluring candidates as genes important in nervous system development and/or function and should be prioritized for further study in worm and mammalian model systems.

In general, our targeted screen of HSA21 orthologs revealed three primary findings for the 10 HSA21 orthologs. First, we found genes with established links to nervous system function (*e.g.*, *cle-1*, *eva-1*, *ncam-1*, and *unc-26*). For instance, *cle-1* (*COL18A1*), *eva-1* (*EVA1C*), and *ncam-1* (*NCAM2*) and have all been linked to deficits in axon guidance in worm. The type XVIII collagen gene, *cle-1*, mediates both neuronal and axonal migrations (Ackley *et al.* 2001). *eva-1* encodes a Slit receptor required for axon guidance, and *ncam-1* encodes a neural cell adhesion molecule that functions redundantly with other cell adhesion molecules to direct axonal outgrowth (Fujisawa *et al.* 2007; Schwarz *et al.* 2009). Additionally, the *unc-26* (*SYNJ*) mutant showed resistance to aldicarb but sensitivity to levamisole indicative of presynaptic defects. This is consistent with its known role in synaptic vesicle recycling (Harris *et al.* 2000).

Second, we uncovered novel neuronal functions for the otherwise well-characterized HSA21 ortholog *rnt-1* (*RUNX1*). In mammals, the runt-related transcription factor *RUNX1* plays a vital role in development, including regulation of the developing nervous system (Wang and Stifani 2017). In worm, the runt-related transcription factor *rnt-1* has also been studied for its role in general development, including cell proliferation and male tail development (Hajduskova *et al.* 2009; Nimmo *et al.* 2005). Interestingly, loss of *rnt-1* may also cause defects in phasmid neuron morphology; however, this was not linked to a detectable behavioral phenotype (Hajduskova *et al.* 2009). The deficiency that the *rnt-1* mutant displayed in our radial dispersion assay might be due to developmental defects affecting the nervous system.

Third, whereas the majority of DS research has focused on well-studied genes, we also uncovered novel neuronal functions of three poorly characterized HSA21 orthologs that have weak or nonexistent links to the nervous system: *dnsn-1* (*DONSON*), *mtq-2* (*N6AMT1*), and *pdxk-1* (*PDXK*). These three genes are so poorly characterized that they only had placeholder names when we started our study. To encourage their study, we renamed them based on their predicted orthologs: *C24H12.5* to *dnsn-1*, *C33C12.9* to *mtq-2*, and *F57C9.1* to *pdxk-1*. Below, we highlight their functions in the limited context of their orthologs in other species.

*DONSON* was only recently found to encode a novel fork protection factor that underlies microcephalic dwarfism, yet it remains deeply understudied (Reynolds *et al.* 2017). The worm ortholog, *dnsn-1*, is equally uncharacterized. We found *dnsn-1* mutants to exhibit extended dwelling in an exploration assay and observed expression of *dnsn-1* in the developing embryo, but not the adult animal. Thus, our data are suggestive of a role within the nervous system and possibly within the developing nervous system.

To our knowledge, MTQ-2 has not been implicated in nervous system function in any animal model. The mammalian ortholog of *mtq-2*, *N6AMT1*, was originally named on the basis of the presence of an amino acid motif (D/N/S)PP(Y/FW) which is characteristic of adenine methyltransferases (Bujnicki and Radlinska 1999; Kusevic *et al.* 2016). However, no evidence of adenine methylation by MTQ-2 protein has been found in either yeast or mouse, suggesting that *N6AMT1* may be a misnomer (Liu *et al.* 2010; Ratel *et al.* 2006). Instead, MTQ-2 has been shown to post-translationally modify ERF1 (eukaryotic release factor 1) by methylating a universally conserved glutamine residue on ERF1 (Heurgué-Hamard *et al.* 2006); (Polevoda *et al.* 2006). More recently, the mouse ortholog of MTQ-2 was shown to methylate many additional substrates *in vitro* and CHD5 (chromodomain helicase DNA-binding protein 5) and NUT (nuclear protein in testis) *in vivo*. This raises the possibility that MTQ-2 may modulate more proteins than previously thought (Kusevic *et al.* 2016). Here we have identified behavioral phenotypes of *mtq-2* mutants that support a neuronal role in *C. elegans*. Additionally, we found that a reporter for *mtq-2* widely expresses throughout the nervous system, specifically in a subset of cholinergic neurons. The high conservation (47% amino acid identity between human isoform 1 and worm) of MTQ-2 as well as many of its predicted interacting partners warrant more detailed study to determine how it functions in synaptic transmission. *C. elegans* may provide an especially informative system to study *mtq-2* as deletion of *N6AMT1* causes embryonic lethality in knockout mice (Liu *et al.* 2010).

PDXK phosphorylates vitamin B6, converting it to PLP (pyridoxal-5′-phosphate), a key cofactor in the metabolism of hundreds of enzymatic reactions, including synthesis of neurotransmitters (Cao *et al.* 2006; Shetty and Gaitonde 1980). Loss of *PDXK* in mice causes pre-weaning lethality, so its behavioral role has not yet been studied in mammals (Mouse Genome Informatics and the International Mouse Phenotyping Consortium). Interestingly, *PDXK* is predicted to be haploinsufficient in mammals and is a candidate dose-sensitive gene contributing to phenotypes in DS (Antonarakis 2016). We found that *pdxk-1* mutant worms showed general deficiencies in behaviors as well as specific defects in synaptic transmission as determined by aldicarb screening. Our finding that the *pdxk-1* mutant was readily paralyzed by levamisole suggests a presynaptic role for *pdxk-1* in the nervous system.

There is a rich body of literature in the field of DS devoted to exploring the underlying genetic contributions of the disorder. Single gene studies have helped to elucidate the role of individual genes such as *DYRK1A*, both as a transgenic single gene triplication and within a trisomic context, and have led to the development of pharmacotherapeutic treatments (Ahn *et al.* 2006; Altafaj *et al.* 2001; Shindoh *et al.* 1996; Song *et al.* 1996). Mouse models have also been instrumental in parsing discrete, chromosomal regions and uncovering genetic pathways that may be amenable to therapeutic targeting (Belichenko *et al.* 2015; Das *et al.* 2013; Jiang *et al.* 2015; Olson *et al.* 2004, 2007). More recently, bioinformatic approaches have been used to explore the transcriptional landscape of DS and highlight candidate genes that may contribute to DS phenotypes (Letourneau *et al.* 2014; Sturgeon *et al.* 2012). Our results provide initial clues on the function of several uncharacterized HSA21 orthologs that add to established findings. Conclusions on the precise mechanism of action of these genes and their possible relevance to DS will benefit from more detailed study in *C. elegans*—including examination of possible phenotypes induced by overexpression—as well as further investigation using traditional mouse models and human cell line approaches.

## Supplementary Material

Supplemental material is available online at www.g3journal.org/lookup/suppl/doi:10.1534/g3.118.200019/-/DC1.
