# Supplementary figures and images for: Systematic Functional Characterization of Human 21st Chromosome Orthologs in Caenorhabditis elegans
Source: G3 (Bethesda). 2018 Jan 24;8(3):967–79. doi: 10.1534/g3.118.200019 (PMC5844316; doi:10.1534/g3.118.200019)

RNAi treatment

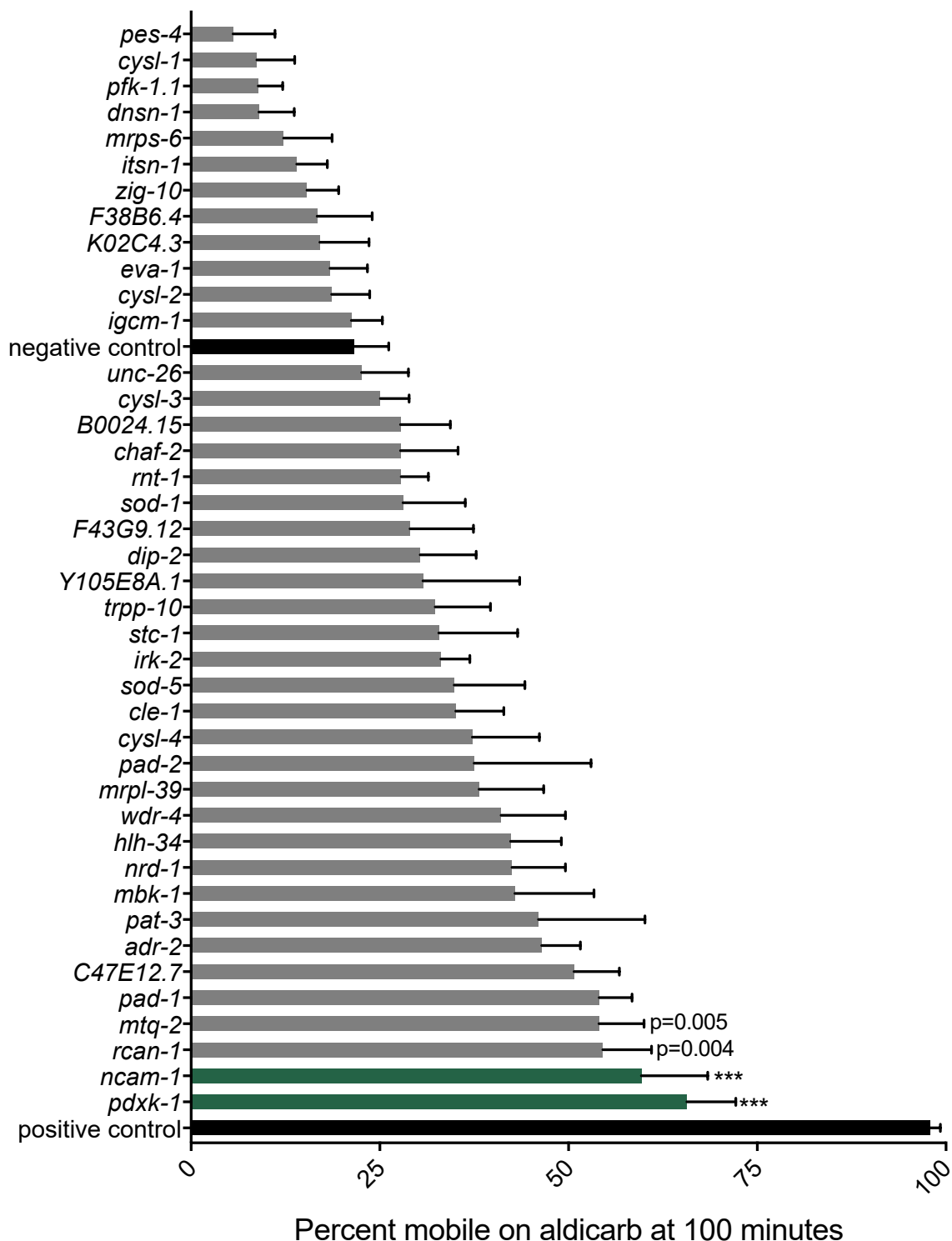

Supplement: Supplementary file 1 [file 967FigureS1.pdf]
